# Infection with *Helicobacter pylori* Is Associated with Protection against Tuberculosis

**DOI:** 10.1371/journal.pone.0008804

**Published:** 2010-01-20

**Authors:** Sharon Perry, Bouke C. de Jong, Jay V. Solnick, Maria de la Luz Sanchez, Shufang Yang, Philana Ling Lin, Lori M. Hansen, Najeeha Talat, Philip C. Hill, Rabia Hussain, Richard A. Adegbola, JoAnne Flynn, Don Canfield, Julie Parsonnet

**Affiliations:** 1 Division of Infectious Diseases and Geographic Medicine, Stanford University School of Medicine, Stanford, California, United States of America; 2 Medical Research Council Laboratories, Fajara, The Gambia; 3 Departments of Microbiology and Molecular Genetics and Immunology, University of Pittsburgh School of Medicine, Pittsburgh, Pennsylvania, United States of America; 4 Departments of Medicine and Microbiology and Immunology, Center for Comparative Medicine, University of California Davis School of Medicine, Davis, California, United States of America; 5 Department of Pediatrics, University of Pittsburgh School of Medicine, Children's Hospital, Pittsburgh, Pennsylvania, United States of America; 6 Department of Pathology and Microbiology, Aga Khan University, Karachi, Pakistan; McGill University, Canada

## Abstract

**Background:**

*Helicobacter pylori*, a lifelong and typically asymptomatic infection of the stomach, profoundly alters gastric immune responses, and may benefit the host in protection against other pathogens. We explored the hypothesis that *H. pylori* contributes to the control of infection with *Mycobacterium tuberculosis*.

**Methodology/Principal Findings:**

We first examined *M. tuberculosis*-specific IFN-γ and *H. pylori* antibody responses in 339 healthy Northern Californians undergoing routine tuberculin skin testing. Of 97 subjects (29%) meeting criteria for latent tuberculosis (TB) infection (LTBI), 45 (46%) were *H. pylori* seropositive. Subjects with LTBI who were *H. pylori*-seropositive had 1.5-fold higher TB antigen-induced IFN-γ responses (*p = 0.04*, ANOVA), and a more Th-1 like cytokine profile in peripheral blood mononuclear cells, compared to those who were *H. pylori* seronegative. To explore an association between *H. pylori* infection and clinical outcome of TB exposure, we evaluated *H. pylori* seroprevalence in baseline samples from two high risk TB case-contact cohorts, and from cynomolgus macaques experimentally challenged with *M. tuberculosis*. Compared to 513 household contacts who did not progress to active disease during a median 24 months follow-up, 120 prevalent TB cases were significantly less likely to be *H. pylori* infected (AOR: 0.55, 95% CI 0.0.36–0.83, *p = 0.005*), though seroprevalence was not significantly different from non-progressors in 37 incident TB cases (AOR: 1.35 [95% CI 0.63–2.9] *p = 0.44*). Cynomolgus macaques with natural *H. pylori* infection were significantly less likely to progress to TB 6 to 8 months after *M. tuberculosis* challenge (RR: 0.31 [95% CI 0.12–0.80], *p = 0.04*).

**Conclusions/Significance:**

*H. pylori* infection may induce bystander effects that modify the risk of active TB in humans and non-human primates. That immunity to TB may be enhanced by exposure to other microbial agents may have important implications for vaccine development and disease control.

## Introduction

The microbes that colonize the human host are diverse and demonstrate geographic and temporal variability. This variability is exemplified by *H. pylori* infection, a gastric mucosal pathogen that comprises part of the “normal flora” in much of the developing world, but has receded over time in higher socioeconomic regions of the world. *H. pylori* has been colonizing humans for at least 50,000 years [1]. Why its prevalence varies so dramatically based on socioeconomic status is not known but may relate to antimicrobial use, improved household and environmental sanitation, and decreased crowding. Another hypothesis, however, is that *H. pylori* infection provides a survival benefit against challenges present disproportionately in poorer geographic regions. By boosting mucosal and systemic immunity, the organism may limit the consequences of other infectious exposures [2], [3] and selectively promote survival of *H. pylori* infected hosts.

One third of the world's population is latently infected with the intracellular pathogen *M. tuberculosis [4]*. Following exposure, the organism elicits an IFN-γ-driven, cellular immune response, causing infected macrophages to be sequestered within organized lung granulomas [5], [6]. In approximately 90% of humans, the host immune response controls but does not eliminate the infection, preventing progression to active disease throughout the host's lifespan [7]. Those infected with *M. tuberculosis* but with no symptoms of disease are referred to as latently infected. Although risk of active tuberculosis is significantly elevated in immunocompromised hosts (e.g., those with HIV infection [8] or treated with immunosuppressants [9]), the great majority of individuals who develop active TB do so in the absence of known immunocompromise. The nature of protective immunity remains unknown.

*M. tuberculosis* and *H. pylori* are the most prevalent bacterial pathogens worldwide. In much of the world's population, these obligate human infections coexist throughout most of the life span, continuously interacting with the host immune system without causing disease. Almost nothing is known about the crosstalk of these infections and whether one infection affects the clinical manifestations of the other. The few studies examining an epidemiologic linkage between *H. pylori* and tuberculosis have yielded conflicting results [10], [11], [12]. While conducting a study of TB diagnostics in a population that had been tested for *H. pylori*, we fortuitously identified differences in interferon gamma (IFN-γ) and other cytokine responses to *M. tuberculosis* antigens in *H. pylori*-infected and uninfected hosts. To explore this relationship further, we compared rates of *H. pylori* seropositivity in blood samples from TB cases and household contacts recruited from TB case-contact studies carried out in The Gambia [13] and Karachi, Pakistan [14]. We also compared outcome of *M. tuberculosis* challenge [15] in macaques with and without naturally-acquired *H. pylori* infection. Our results support further investigations into the contribution of *H. pylori* infection to the protective immune response to TB infection.

## Materials and Methods

### Overview

Three distinct studies were undertaken sequentially. Studies involving human subjects or samples were conducted in accordance with principles expressed in the Declaration of Helsinki. Non-human primate studies were conducted in accordance with the United States Animal Welfare Act and the Guide for the Care and Use of Laboratory Animals of the Institute for Laboratory Animal Research, National Academies of Science. Each study was approved by the appropriate Institutional Review Boards as described.

#### (1) IFN-γ responses to TB antigens in Northern Californians with and without *H. pylori* infection

The Stanford Infection and Family Transmission [SIFT] study was established in 1999 to evaluate incidence of *H. pylori* infection within predominately immigrant communities of the South Peninsula, San Francisco Bay. Since 2003, we have tested concurrently for latent *M. tuberculosis* infection. Data used in this report include 339 healthy residents of Santa Clara County, CA who gave written consent between September 2003 and May 2006 to provide blood for QuantiFERON-TB GOLD (in-tube) IFN-γ release assay (Cellestis, Ltd, Melbourne, Australia), as well as for *H. pylori* and other infectious disease testing, at the time of routine tuberculin skin test (***Table 1***). Children under 2 years of age, and individuals with history or symptoms of active tuberculosis were not recruited. The population is predominately Hispanic, including 50% born in a TB endemic country (90% Latin America). The study was approved by the Institutional Review Boards of Stanford University and the Santa Clara Valley Medical Center.

**Table 1:** Population characteristics: Northern California *H. pylori*/LTBI studies.

Characteristic	Total (n = 339)	*H. pylori*+ (n = 101)	*H. pylori*− (n = 238)
Age, mean, range	28 (3–80)	31 (8–80)	27 (3–75)
2–17 y	113 (33)	21 (21)	92 (39)
≥18y	226 (67)	80 (78)	146 (61)
Sex			
Male	126 (37)	48 (48)	78 (33)
Female	213 (63)	53 (52)	160 (67)
Hispanic ethnicity	238 (70)	90 (89)	148 (62)
Foreign-born^1^	167 (49)	73 (72)	94 (40)
Hepatitis A total IgG	212 (63)	79 (78)	133 (56)
Mycobacterial infections/exposures			
BCG scar (present)	114 (34)	47 (47)	67 (28)
History TB exposure	46 (14)	20 (20)	26 (11)
Latent TB infection^2^			
TST+	82 (24)	40 (40)	42 (18)
QFT+	48 (14)	23 (24)	25 (11)
*Either+*	97 (29)	45 (45)	52 (22)

Characteristics of healthy individuals referred through public health clinics in Santa Clara County, CA, USA who completed tuberculin skin test [TST], QuantiFERON-TB GOLD® interferon-γ release assay, and *H. pylori* serology.

1 152 (91%) born in Latin America; TST: tuberculin skin test, +, ≥ 10mm induration (includes 18 prior positives not retested); QFT (QuantiFERON-TB-GOLD® including *M. tuberculosis* antigens ESAT6 and CFP10 and *M. tuberculosis* antigen TB7.7; +: ≥0.35 IU/ml IFN-γ difference over unstimulated well.

2 Latent TB infection [LTBI]: either TST+ or QFT+ for analysis; 7 (2%) individuals reported prophylactic TB treatment 3–34 years prior to enrollment. All factors significant at p<0.05 (2-sided χ^2^ test).

#### (2) *H. pylori* sero-prevalence in human tuberculosis case-contact cohorts

De-identified plasma samples obtained at a baseline screening visit were recruited from the specimen banks of tuberculosis case-contact studies conducted by the Medical Research Council, The Gambia, West Africa [16] and the Aga Khan University, Karachi, Pakistan [14], respectively. Each study enrolled households based on an index case of active tuberculosis, and assessed participants clinically for at least 24 months from baseline, with overall rates of activation 1.1% (The Gambia [13]) and 6.4% (Karachi [14]) previously reported. Active TB was ascertained by symptoms, chest X-ray and AFB smear and culture in The Gambia [16], and by symptoms, chest X-ray and AFB smear in Pakistan [14]. Baseline TB infection was determined by positive (≥10 mm) TST and/or ELISPOT in The Gambia [17] and by positive TST (≥10 mm in Pakistan [14]. Blood samples recruited for the present studies were from HIV-negative subjects with blood drawn before the onset of treatment of a TB prevalent or incident case.

**Figure 1:**
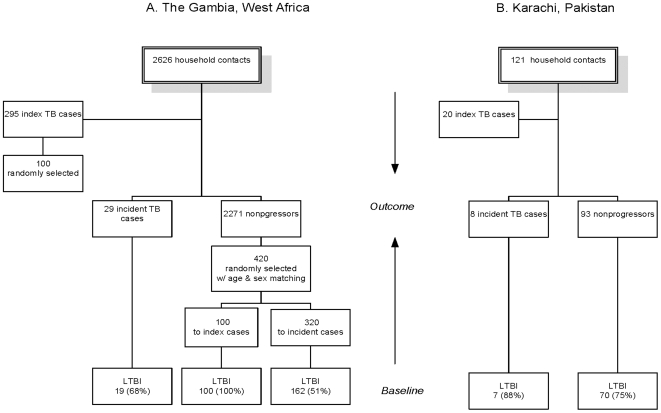
Samples recruited for *H. pylori* testing from Tuberculosis Case-Contact Studies. Blood samples were recruited for *H. pylori* serology testing from the specimen banks of tuberculosis case- contact studies carried out in (**A**) The Gambia, West Africa (Ref. 13,16), and (**B**) Karachi, Pakistan (Ref 14). Each study followed household members exposed to an active (index) case of tuberculosis for at least 2 years. Eligible samples were from HIV-negative participants. Samples from Gambia were randomly selected in 2 groups to match household contacts by age and sex to index and incident TB cases, respectively. *LTBI*: latent tuberculosis infection at baseline, defined as TST ≥10mm or ELISPOT ≥8 SFU (Gambia), or as TST ≥10mm (Pakistan). Baseline characteristics are shown in Table S4.

Sampling proportions and baseline characteristics of subjects whose samples were selected for analysis are shown in Table S4. From The Gambia, 549 samples were randomly selected in 2 phases from a pool of 2626 eligible samples (***Figure 1A***). The first group consisted of samples from 100/295 eligibleTB cases (household index cases) and 100/2271 eligible age- and sex-matched household contacts who were TB infected at baseline and known to have remained disease-free for at least 2 years of follow-up (nonprogressors). Samples from Gambian TB cases were proportionately representative of Gambian TB isolates (approximately 40% *M. africanum* and 60% *M. tuberculosis* [18]). The second sampling phase included samples from 29/32 subjects who developed active TB 3–47 months from baseline, and 320 age- and sex-matched nonprogressors. The study was approved by the Institutional Review Boards of Stanford University and the MRC Ethics Committee. A complete sample was obtained from the Pakistan cohort (***Figure 1B***), including 20 index TB cases, 8 incident TB cases ascertained 3–49 months from baseline, and 93 household contacts who remained disease-free. The study was approved by the Institutional Review Boards of Stanford University and the Aga Khan University Ethics Committee.

#### (3) TB challenge in cynomolgus macaques with and without naturally acquired *H. pylori* infection

Stored baseline sera, frozen gastric necropsy samples, and clinical necropsy reports were obtained from a convenience sample of 45 cynomolgus macaques (*Macaca fasicularis*) (>4 years of age) used in ongoing or completed low-dose TB challenge studies. All monkeys included in our study had been challenged endotracheally with 25 CFU of the Erdman strain of *M. tuberculosis*. Monkeys were assessed clinically for at least eight months post-challenge, and were classified as having active TB or latent infection based on previously defined criteria [19]. Specifically, monkeys were classified as having active TB if they had any clinical signs of disease, including weight loss, appetite loss, cough, radiographic findings, elevated erythrocyte sedimentation rate (ESR), or BAL or gastric aspirate that grew *M. tuberculosis* after the first two months of infection. Monkeys were classified as latent if they exhibited no signs of active disease (by the measures described above) and had culture negative BAL and gastric aspirate samples after 2 months of infection. These criteria have been validated at necropsy in a large group of monkeys [20]. The Pittsburgh model has previously yielded approximately 50% active and 50% latent disease post-challenge [19], [20]. The outcome is independent of gender, weight or age (between 4–10 years of age) in this model [20]. The animals were housed and maintained in accordance with standards established in the Animal Welfare Act and the Guide for the Care and Use of Laboratory Animals. All procedures and protocols were approved by the Institutional Animal Care and Use Committee (IACUC) of the University of Pittsburgh.

### Laboratory Methods

#### *H. pylori* infection in humans

The diagnosis of *H. pylori* infection was ascertained by enzyme-linked immunosorbent assay (ELISA). Per protocol, all samples were run in triplicate by technicians blinded to the TB infection or outcome classification of samples, and samples with different infection classifications were intermixed on the same plates and run on the same days. Final serologic interpretations were completed before samples were linked to case records.

For the Northern California subjects, a high molecular weight whole cell lysate ELISA antigen was derived from 2 Asian, 2 Mexican, and 2 US isolates of *H. pylori* with results interpreted according to validated cut-offs, as previously described [2], [21]. Because antibodies to *H. pylori's* immunodominant CagA protein, present in approximately 65% of *H. pylori* isolates, may be more reliable in samples from developing countries [22], [23], Pakistan and Gambian samples were tested for IgG antibody response to the orv220 fragment of *H. pylori* CagA (courtesy H. Kleanthous) as previously described [24]. A sample was considered positive if optical density reading exceeded the mean + 3SD of 15 CagA negative controls. Indeterminate samples (n = 24) were classified as negative for analysis.

#### Measles antibodies

To establish baseline humoral response, quantitative IgG antibody response to measles was compared in a subset of samples from 100 culture-confirmed Gambian TB patients and 100 age and sex-matched household contacts with latent infection, using the Bioquant IgG ELISA (San Diego, CA) with qualitative results interpreted according to manufacturer's criteria.

#### TB antigen-induced cytokine studies

We used the QuantiFERON-TB-GOLD® (Cellestis, Ltd, Carnegie, Victoria, Aus) assay to quantify stimulated PBMC IFN-γ responses to specific *M. tuberculosis* peptides as previously described [25]. Quantitative results, expressed as IU/ml, representing the difference in IFN-γ response to *M. tuberculosis* peptides or PHA and a saline control for each subject, were transformed for statistical analysis using the natural log base. Residual supernatants from stimulated PBMCs were stored at −80°C and subsequently, 65 samples from adults were further assayed in a Luminex xMAP 200 platform using customized bead preparations (Beadlyte Human 26-plex Multi-Cytokine, Millipore, Billerica, MA). Concentrations for a standard reference curve were included on each plate per manufacturer's instructions. For each subject cytokine concentrations in the unstimulated control well were subtracted from TB specific responses, expressed as pg/ml.

#### *H. pylori* diagnostic procedures in monkeys

Frozen serum samples obtained before *M. tuberculosis* challenge (N = 44) or soon thereafter (1 sample obtained 33 days post infection) were sent to Stanford University for ELISA. A rhesus-derived *H. pylori* strain was employed for the ELISA antigen. In prior studies with the rhesus model, this assay had an estimated sensitivity and specificity against endoscopy or breath test of 96% and 88% respectively [26]. Gastric tissue obtained at necropsy (n = 28 monkeys) and frozen at −80C for between 1 week and 22 months (median 13.8 months), was shipped to U.C. Davis, fixed, and processed in the laboratory of J. Solnick for *H. pylori* culture and histopathology respectively as previously described [15].

### Statistical Analysis

Statistical analysis was conducted using SAS v. 9.3. All statistical tests were carried out at alpha set to 0.05 for a two-sided test. The following analyses were conducted:

#### (1) IFN-γ responses to TB antigens in Northern Californians with and without *H. pylori* infection

To evaluate whether concurrent *H. pylori* infection affects the immune response to *M. tuberculosis* antigens, we compared quantitative TB specific IFN-γ responses stratified by *H. pylori-* and latent *M. tuberculosis* infection. *H. pylori* infection was defined by results of the whole cell lysate ELISA. We defined latent TB infection (LTBI) presumptively as either *M. tuberculosis* specific IFN-γ response ≥0.35 IU/ml or TST result ≥10 mm, with no signs or symptoms of active TB. A Generalized Linear Model was used to compare group means accounting for the interaction of *H. pylori* and LTBI as well as to adjust for potential confounders (age, sex, and country of origin); p-values are based on the F-test (Fisher's LSD) for pre-planned contrasts (e.g. *H. pylori* effect within LTBI). For multiplex-cytokine analysis, Principal Components Analysis [27] was performed with the correlation matrix composed of six variables found to discriminate by Wilcoxon's test between 40 latently infected and 25 controls (*Tables S1* and *S2*), and components having Eigenvalue >1 were selected to compute summary component scores for each subject using eigenvector coefficients.

#### Comparison of *H. pylori* prevalence in human tuberculosis case-contact cohorts

We compared rates of *H. pylori* CagA infection in index TB cases, incident TB cases, and household contacts known not to have progressed to active disease during follow-up (nonprogressors). Logistic regression was used to evaluate factors associated with *H. pylori* infection in each cohort and in the cohorts combined. Odds ratios and 95% confidence intervals of *H. pylori* infection in nonprogressors compared to prevalent or incident TB cases were computed with adjustment for age, gender, latent *M. tuberculosis* infection at baseline, and cohort location (Pakistan or The Gambia). Goodness of fit was evaluated using the Hosmer-Lemeshow test [28]. A generalized nonlinear mixed model with random intercept (SAS GLIMMIX) was used to examine results with adjustment for household membership.

#### *M. tuberculosis* challenge in cynomolgus macaques with and without naturally acquired *H. pylori* infection

Monkeys were categorized clinically by 6–8 months after aerosol infection as having active tuberculosis or latent infection. *H. pylori* infection in monkeys was defined as a positive serologic test for *H. pylori* prior to M. tuberculosis infection. Because gastric tissue had not been obtained or conserved for the purposes of *H. pylori* histology or culture, negative results to these two tests were not considered reliable for determining presence or absence of infection. Relative risk and 95% confidence intervals of active vs. latent tuberculosis in *H. pylori* infected versus uninfected monkeys was computed.

## Results

### IFN-γ Responses to TB Antigens in Northern Californians with Concurrent *H. pylori* Infection

Of 339 subjects (113 children and 226 adults), 97 (29%) individuals met criteria for latent TB infection, including 82 (24%) individuals with TST induration ≥10mm and 48 individuals (14%) with TB specific antigen induced IFN-γ ≥0.35 IU/ml. A total 242 (71%) individuals were negative by both criteria and classified as not latently infected. Of subjects classified as latently infected, 45 (46%) were *H. pylori* seropositive as compared with 52 (22%) of the 242 subjects classified as LTBI negative (p<0.001).

**Figure 2:**
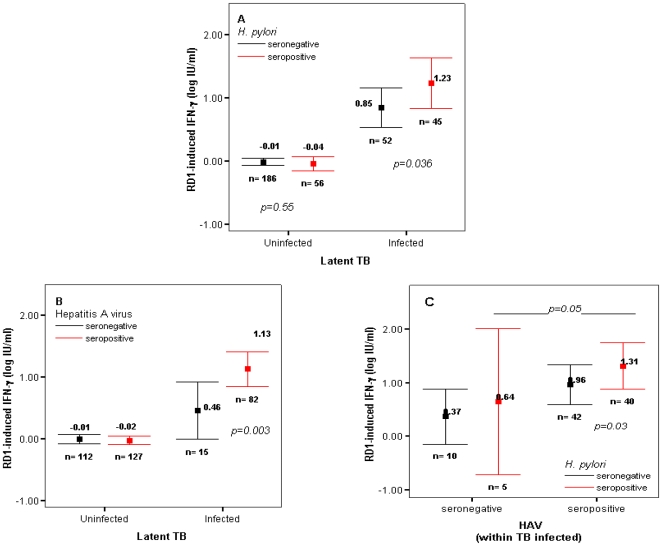
IFN*-γ* responses to TB antigens are amplified in concurrently infected subjects. **A**: TB antigen induced (24 hour) IFN-γ responses in 97 subjects classified as TB infected [LTBI] and 242 subjects classified as without LTBI, stratified by *H. pylori* infection status; *p-values* denote age and sex-adjusted difference within LTBI classification (ANOVA). **B.** panel A obtained by substituting results of Hepatitis A virus total IgG antibody response [HAV] for *H. pylori* response; **C.** mean IFN-γ levels within 97 LTBI+ by *H. pylori* and HAV antibody test results; *p-values* denote contrast within each infection grouping. LTBI+: TST≥10 mm induration or QuantiFERON-TB GOLD® positive (≥0.35 IU/ml difference over unstimulated well); LTBI−: TST<10mm and QuantiFERON-TB GOLD®-<0.35 IU/ml; *error bars* represent 95% confidence interval of the least squares means.

Compared to 52 latently infected subjects without *H. pylori* infection, mean IFN-γ responses to specific TB antigens were approximately 1.5 fold greater in 46 subjects with concurrent *H. pylori* infection (***Figure 2A***), and this difference remained significant after adjusting for age, sex and country of birth (*p = 0.04*, ANOVA). In contrast, IFN-γ responses did not vary significantly with respect to *H. pylori* infection among 242 LTBI-negatives (*p =  0.40*), indicating an interaction effect between *H. pylori* and LTBI (*p = 0.03*, *ANOVA*). These results were similar when restricting the definition of LTBI to TST positivity only (*data not shown*).

The effect on IFN-γ responses to *M. tuberculosis* antigens of past exposure to Hepatitis A virus, a picornavirus infection with epidemiologic patterns similar to those of *H. pylori* [29] was similar to that for *H. pylori (* ***Figure 2B*** *)*. In fact, in a 3-way interaction model, having *H. pylori* infection, HAV and LTBI was associated with greater IFN-γ responses to TB than having either infection singly with LTBI (***Figure 2C***).

**Figure 3:**
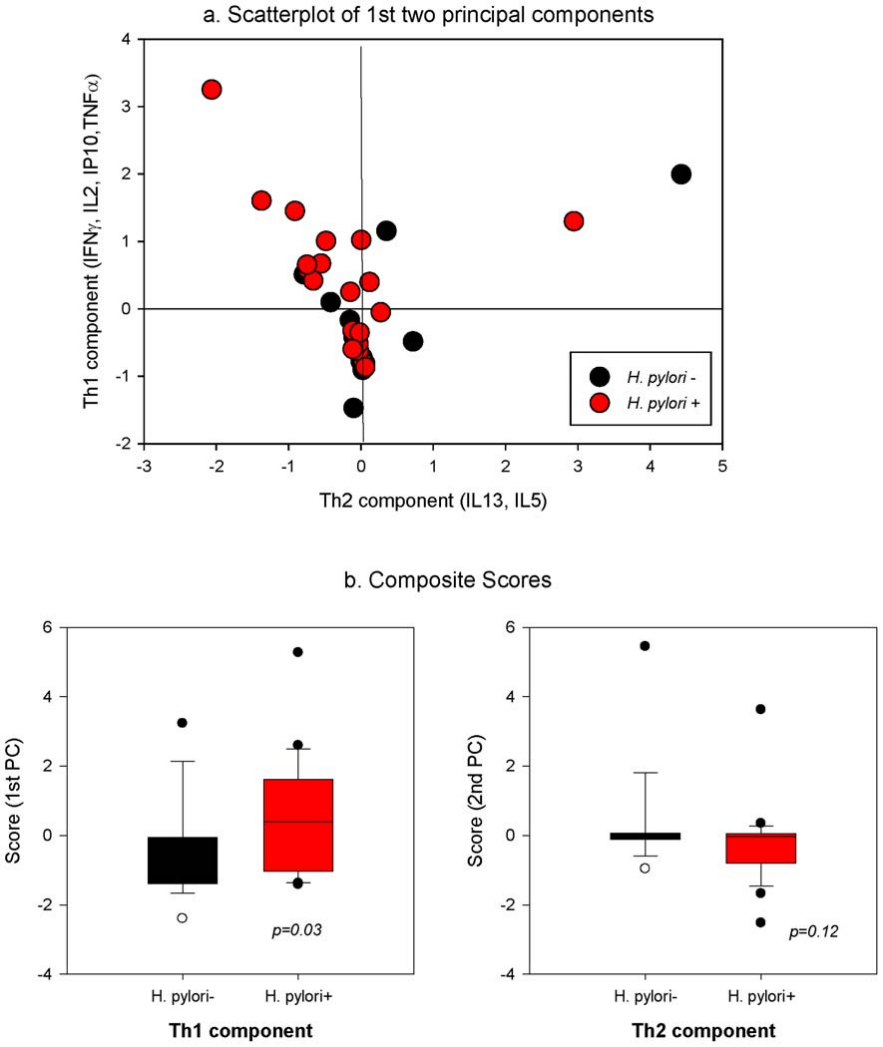
Concurrent *H. pylori* infection is associated with Th1^+^/Th2^−^ type profile. **A.** Scatterplot of principal component scores for 40 adults with latent TB infection (either QuantiFERON-TB GOLD® or TST positive) obtained after linear transformation with eigenvector coefficients. *Vertical-axis*: 1^st^ component (eigenvalue 2.6), which is largely explained by IFN-γ and IL-2, and to a lesser extent IP-10 and TNF-α. *Horizontal axis:* 2nd PC (eigenvalue 1.5), characterized by loading of IL-13 and IL-5 and negative correlations with the four inflammatory markers. *Red circles:* 23 *H. pylori* infected; *Black circles:* 17 *H. pylori* seronegative. Reference lines denote quadrants centered on 0; **B.** comparison of median composite Th1 and Th2 component scores; *p-values* computed by Wilcoxon's 2-sided median test.

In the 65 adults (40 with LTBI and 25 without) tested by 26-multiplex cytokine assay (*Table S1*), IL2, TNF-α, CXCL-10 (IP10), IL-13 and IL-5, as well as IFN-γ, were differentially detected in latently infected vs. uninfected subjects (*Table S2*). Within the 40 latently infected, samples from the 23 subjects with concurrent *H. pylori* infection had higher summary measures of Th-1-type (IFN-γ, IL2, TNF-α, CXCL-10) cytokine responses to TB antigens (*p = 0.03*, Wilcoxon) than did the 17 *H. pylori* seronegative (***Figure 3a and b*** *; Table S3*). Overall, 9 (82%) of 11 subjects classified as having dominant Th1-type cytokine responses to *M. tuberculosis* infection were *H. pylori* seropositive (*p = 0.05* Fishers exact test).

### Comparison of *H. pylori* Sero-Prevalence in Human Tuberculosis Case-Contact Cohorts

Among a total 670 samples tested, 425 (63%) were *H. pylori* seropositive according to the CagA assay, including 59/121 (49%) of Karachi, Pakistan and 366/549 (67%) of Gambian household contacts (*p = 0.0002*, *supplemental Table S5*). *H. pylori* seroprevalence varied significantly by TB outcome classification (***Table 2***, and *Table S6* for results by cohort). Compared to 513 household contacts who remained disease-free throughout follow-up, the 120 TB cases were about ½ as likely to be *H. pylori* CagA positive (AOR: 0.55 (95% CI 0.36–0.83, *p = 0.005*), while seroprevalence was not different between nonprogressors and 37 incident TB cases (AOR: 1.35 [95% CI 0.65–3.0), *p = 0.44*). These results were similar when restricted to samples from 466 subjects considered latently infected, as well as when accounting for household membership in a random intercept nonlinear mixed model.

**Table 2:** Factors associated with *H. pylori* CagA infection in tuberculosis case-contact cohort samples (*adjusted odds ratio*).

Factor	Reference	All samples (n = 670)	*p-value*	Latent TB (n = 466)	*p-value*
Age	10 y	0.87 (0.79–0.97)	*0.01*	0.93 (0.83–1.1)	*0.26*
Sex	Female	0.97 (0.70–1.4)	*0.88*	0.94 (0.64–1.4)	*0.74*
LTBI	Uninfected	0.79 (0.55–1.2)	*0.22*		
**TB Outcome**	Nonprogressor	1.0		1.0	
	Prevalent (Index) Case	0.55 (0.36–0.83)	*0.005*	0.51 (0.32–0.8)	*0.003*
	Incident TB case	1.35 (0.63–2.9)	*0.44*	1.3 (0.53–3.2)	*0.56*
Pakistan TBCC	Gambia	0.46 (0.31–0.69)	*<0.001*	0.37 (0.23–0.59)	*<0.001*
	*H-L χ^2^ 10.02 (p = 0.25)*			*H.L.χ^2^ 3.6 (p = 0.91)*	

*Nonprogressor*: household contact of TB case remaining disease free at least 2 years from baseline; *Prevalent (Index) case*: untreated active TB case at baseline; *Incident TB case:* household contact developing active TB 3–49 months from baseline (*see methods).; H.L.:* Hosmer-Lemeshow test.

To explore whether the diminished antibody responses to *H. pylori* in prevalent TB patients might result from immune deficits associated with having active disease, we evaluated measles antibody responses in 100 Gambian culture-confirmed TB cases and 100 age- and sex-matched latently infected nonprogressors. Equal numbers of cases and contacts responded to a measles IgG antibody test (90% and 88% respectively) with virtually identical mean optical density values (*Figure S1*), suggesting that active TB disease did not cause a diminution of humoral responses.

### TB Challenge in Cynomolgus Macaques with and without Naturally Acquired *H. pylori* Infection

Of the 45 monkeys, 4 monkeys had indeterminate *H. pylori* results by the rhesus-based *H. pylori* ELISA and were excluded from the analysis. Among the remaining 41 monkeys, 30 (73.2%) were seropositive and 11 (26.8%) were seronegative for *H. pylori*. Although, as expected, culture yields in seropositive monkeys were low due to tissue autolysis and storage, all 6 *H. pylori* culture-positive monkeys were positive by serology, as were 4 of 6 *H. pylori* histology positive monkeys. Of the 30 monkeys with positive *H. pylori* serology (***Table 3***), only 5 (16.7%) developed active tuberculosis whereas 6 (54.6%) of the 11 negative monkeys developed active tuberculosis (relative risk 0.31, 95% CI 0.12–0.80, *p = 0.04*, Fisher's Exact test).

**Table 3:** Seroprevalence of *H. pylori* infection in 41 Pittsburgh cynomolgus macaques by outcome of TB challenge at 6–8 months.

*H. pylori* infection	Active TB	Latent TB	Total
Infected	5 (17)	25 (83)	30 (73)
Uninfected	6 (55)	5 (45)	11 (27)
Total	11	30	41
**Relative risk of active TB in ** ***H. pylori*** ** infected: 0.31 (0.12–0.80)** *p = 0.04 (Fisher's exact test)*			

## Discussion

Humans are colonized by complex, site-specific microbial communities that are increasingly implicated in human health and disease in unexpected ways. In some cases, microbial communities are thought to modulate non-infectious diseases, such as obesity [30], asthma and autoimmune diseases [31]. Viewed broadly, the human microbiota may be a major regulator of the immune system modulating not only inflammatory disorders (the “hygiene hypothesis”), but responses to infectious challenges as well [32]. Because they continuously stimulate non-specific responses in the host, chronic mucosal infections may be particularly important in this regard.

The high prevalence of *H. pylori* in populations where TB and other lethal infections remain endemic suggests the host-pathogen interplay of these infections has co-evolved. In this report, we have presented evidence from three different study designs that *H. pylori* infection affects response to *M. tuberculosis*. First, we found in a cross-sectional human study that *H. pylori* is associated with enhanced IFN-γ and Th1-like responses to specific TB antigens. Second, in baseline samples from two high risk human tuberculosis case-contact cohorts, we observed that infected household contacts who maintain latency over two years are significantly more likely to have concurrent *H. pylori* infection than are TB cases, although seroprevalence was not different in incident TB cases. Finally, in a retrospective cohort study of low-dose *M. tuberculosis* challenge monkeys, we observed that *H. pylori*-infected animals are significantly less likely to develop active disease than are uninfected animals. While further studies are indicated, taken together, these lines of investigation offer a heretofore unexplored role for infections like *H. pylori* to alter the outcome of *M. tuberculosis* infection.

We propose two potential models by which *H. pylori* could promote a protective immune response to TB infection. In the first model, an infection in early childhood could permanently differentiate immature T-cells to a Th1-like phenotype. Such a “hygiene hypothesis,” has been advanced for hepatitis A [33], and would favor past as well as ongoing infections contributing to a state of heightened immunity. Alternatively, *H. pylori* might induce a bystander effect with continuous inflammation and T-cell signaling enhancing the host's innate response to a spectrum of infectious challenges. Similar effects have been demonstrated with γ-herpesvirus in mice challenged with *Listeria monocytogenes* and *Yersinia pestis* [34]. These authors speculate that non- specific induction of interferon-γ (IFN-γ) activates macrophages and primes innate defenses to other infections. We did not test for antibodies to HAV or herpesviridae in the human tuberculosis case-contact cohorts. Because these exposures are likely to be very common in populations where both *H. pylori* and *M. tuberculosis* infections are common, more systematic sampling designs may be needed to adequately explore interaction effects.

*H. pylori* naturally infects macaques. The fact that we were able to replicate a significant association of *H. pylori* infection with latent outcomes in the Pittsburgh cynomolgus monkey TB challenge model [19], [35] offers new opportunities to explore mechanistic arguments in depth. Because the present work is based on a convenience sample from the Pittsburgh laboratory, repeated measures of immunologic responses and clinical progression were not investigated. More systematic experimental study designs, including prospective studies utilizing *H. pylori* challenge, are planned.

The accuracy of *H. pylori* serology in the developing world is suboptimal [22]. Although the CagA assay may be more reliable for comparing different populations, misclassification of *H. pylori* infection status still cannot be excluded. Typically, misclassification tends to yield a null result unless, for some reason, cases and controls respond differently to the assay. We speculated that TB cases might have weakened antibody responses, causing us to have a spurious result. However, the fact that measles antibody titers were robust and virtually the same in Gambian TB cases and age and sex-matched latently infected contacts argues against this explanation of results. That *H. pylori* seroprevalence did not differ between incident TB cases and nonprogressors in our cohort samples, while limiting our conclusions, can also reflect artifacts of the TBCC study model, including the low numbers of secondary cases, ascertainment differences or biases, as well as limitations of *H. pylori* serologic detection in this setting. In both cohorts, most cases of TB were identified at an initial screening visit, and *M. tuberculosis* infection at baseline was a relatively weak predictor of progression [14], [36]. Thus, continued work with longitudinal cohorts may benefit from multi-site study designs incorporating additional *H. pylori* diagnostics as well as other prognostic biomarkers of immune response to TB exposure and infection.

*M. tuberculosis* specific antigen interferon-γ release assays are important noninvasive tools for measuring immunogenicity of the heterologous prime boost T-cell based vaccines for TB [37], [38]. Our results raise the possibility that *H. pylori* infection and other Th-1 modifying infections present in the host background can alter these profiles. As our results also do not exclude the possibility that *M. tuberculosis* modifies the host response to *H. pylori* infection, PBMC responses to TB antigens pre- and post- antibiotic treatment for *H. pylori* would help shed light on the specificity of our findings, including reversibility. *In vitro* studies examining possible mechanisms of T-cell cross reactivity, such as effect of *H. pylori* antigen on *M. tuberculosis* antigen presenting cells and MHC expression, are also needed.

Why only 10% of infected individuals succumb to tuberculosis remains one of the most vexing public health questions–one which the one-pathogen-one-disease paradigm is ill-equipped to answer. While preliminary, our work suggests that one factor contributing to the clinical outcome of TB infection may be a concurrent chronic infection. The hypothesis that the human microbiome has evolved to provide context-specific competitive risk advantages to the host [39] also raises the intriguing possibility that our microbiota can be manipulated to modulate disease risk from *M. tuberculosis*, as well as other common human pathogens [40].

## Supporting Information

Table S1 Characteristics of samples selected for multiple cytokine panels (Northern California study). ^1,2^LTBI: latent tuberculosis infection; +, QuantiFERON [QFT]-TB GOLD® or tuberculin skin test (≥10mm) positive; −, QuantiFERON-TB GOLD® negative and tuberculin skin test negative (<10 mm induration). *** p<0.01, ** 0.01≤p<0.05, *0.05≤p<0.10 vs. unselected. p-values calculated by Chi square or Fisher's exact test.
(0.04 MB DOC)

Table S2 Whole blood TB antigen induced cytokine concentrations (pg/ml) in 65/225 adults. LTBI: latent tuberculosis infection; +, QuantiFERON-TB GOLD® or tuberculin skin test (≥10mm) positive; −, QuantiFERON-TB GOLD® negative and tuberculin skin test negative (<10 mm induration). IQR, interquartile range. % responding, based on proportion of difference values below or above extrapolation limits of a 5 parameter logistic curve for each analyte.
(0.04 MB DOC)

Table S3 Results of principal components analysis performed with six variables (TB antigen-induced cytokine/chemokine results selected to discriminate between latently infected adults and negative controls). Components with Eigenvalues >1 are shown.
(0.04 MB DOC)

Table S4 Baseline characteristics of human cohort samples. LTBI: latent tuberculosis infection determined by TST ≥10mm and/or ELISPOT ≥8 SFU (Gambia) or TST ≥10 mm (Pakistan).
(0.04 MB DOC)

Table S5 Seroprevalence of *H. pylori* CagA infection in TB cases and household contacts: differences between The Gambia and Pakistan.
(0.03 MB DOC)

Table S6 Factors associated with *H. pylori* CagA infection in Gambia and Pakistan tuberculosis case-contact cohort samples (univariate analysis).
(0.04 MB DOC)

Figure S1 Measles antibody responses in 100 Gambian TB cases and 100 latently infected household contacts. *Ref. line*: positive cut-off (Bioquant IgG ELISA, San Diego, CA); Nonprogressor, household contact of TB index case remaining disease-free for at least 2 years from baseline; TB infected, positive TST ≥10mm or ELISPOT ≥8 SFU at baseline.
(0.04 MB DOC)
