# Supplementary material for: Infection with Helicobacter pylori Is Associated with Protection against Tuberculosis
Source: PLoS One. 2010 Jan 20;5(1):e8804. doi: 10.1371/journal.pone.0008804 (PMC2808360; doi:10.1371/journal.pone.0008804)
Supplement: Table S1 — Characteristics of samples selected for multiple cytokine panels (Northern California study). 1,2LTBI: latent tuberculosis infection; +, QuantiFERON [QFT]-TB GOLD® or tuberculin skin test (≥10mm) positive; −, QuantiFERON-TB GOLD® negative and tuberculin skin test negative (<10 mm induration). *** p<0.01, ** 0.01≤p<0.05, *0.05≤p<0.10 vs. unselected. p-values calculated by Chi square or Fisher's exact test. (0.04 MB DOC) [file pone.0008804.s001.doc]

**Supplemental Table 1**

**Table S1. Characteristics of samples selected for multiple cytokine panels (Northern California study)**

| Characteristic | LTBI-negative^1^ | | LTBI-positive^2^ | |
| --- | --- | --- | --- | --- |
| *Number (%)* | All adults  (n=154) | Sampled  (n=25) | All adults  (n=72) | Sampled  (n=40) |
| Male | 39 (25) | 13 (52)*** | 30 (42) | 17 (43) |
| BCG scar | 28 (18) | 5 (20) | 39 (54) | 23 (58) |
| Foreign-born | 54 (35) | 7 (28) | 60 (83) | 39 (98)*** |
| TB exposure, ever | 24 (16) | 6 (24) | 17 (24) | 8 (20) |
| *H. pylori* seropositive | 41 (27) | 10 (40)* | 39 (54) | 23 (58) |
| Hepatitis A total IgG | 71 (47) | 10 (40) | 64 (89) | 40 (100)*** |

^1,2^LTBI: latent tuberculosis infection; +, QuantiFERON [QFT]-TB GOLD® or tuberculin skin test (≥10mm) positive; −, QuantiFERON-TB GOLD® negative and tuberculin skin test negative (<10 mm induration). *** p<0.01, ** 0.01≤p<0.05, *0.05≤p<0.10 vs. unselected . *p-values* calculated by Chi square or Fisher’s exact test.
