# Supplementary material for: Infection with Helicobacter pylori Is Associated with Protection against Tuberculosis
Source: PLoS One. 2010 Jan 20;5(1):e8804. doi: 10.1371/journal.pone.0008804 (PMC2808360; doi:10.1371/journal.pone.0008804)
Supplement: Table S2 — Whole blood TB antigen induced cytokine concentrations (pg/ml) in 65/225 adults. LTBI: latent tuberculosis infection; +, QuantiFERON-TB GOLD® or tuberculin skin test (≥10mm) positive; −, QuantiFERON-TB GOLD® negative and tuberculin skin test negative (<10 mm induration). IQR, interquartile range. % responding, based on proportion of difference values below or above extrapolation limits of a 5 parameter logistic curve for each analyte. (0.04 MB DOC) [file pone.0008804.s002.doc]

**Supplemental Table 2**

**Table S2. Whole blood TB antigen induced cytokine concentrations (pg/ml) in 65/225 adults**

**selected from the Northern California series (40 LTBI+ and 25 LTBI−).**

|  | **IFN-γ** | | **IL-2** | | **IP-10** | | **TNF-α** | | **IL-13** | | **IL-5** | |
| --- | --- | --- | --- | --- | --- | --- | --- | --- | --- | --- | --- | --- |
|  | *LTBI+* | *LTBI-* | *LTBI+* | *LTBI-* | *LTBI+* | *LTBI-* | *LTBI+* | *LTBI-* | *LTBI+* | *LTBI-* | *LTBI+* | *LTBI-* |
| Median | 57.3 | 0 | 113.8 | 0 | 8441 | 143 | 10.4 | -25 | 0 | 0 | 0 | 0.04 |
| IQR | 180.9 | 1.65 | 349.2 | 0 | 13,978 | 872 | 58.4 | 40.7 | 40.3 | 0 | 1.2 | 0.2 |
| Maximum | 648.1 | 47.7 | 2730 | 15.6 | 27,852 | 9380 | 273.9 | 45.5 | 351.3 | 47.0 | 52.0 | 3.5 |
| *p-value (Wilcoxon)* | *<0.0001* | | *<0.0001* | | *<0.0001* | | *0.0007* | | *0.001* | | *0.20* | |
| %  Responding | 88 | 48 | 75 | 16 | 88 | 64 | 58 | 16 | 48 | 8 | 48 | 56 |

*LTBI*: latent tuberculosis infection;  *+*, QuantiFERON-TB GOLD® or tuberculin skin test (≥10mm) positive; −, QuantiFERON-TB GOLD® negative and tuberculin skin test negative (<10 mm induration). IQR, interquartile range. *% responding*, based on proportion of difference values below or above extrapolation limits of a 5 parameter logistic curve for each analyte.
