# Supplementary material for: Infection with Helicobacter pylori Is Associated with Protection against Tuberculosis
Source: PLoS One. 2010 Jan 20;5(1):e8804. doi: 10.1371/journal.pone.0008804 (PMC2808360; doi:10.1371/journal.pone.0008804)
Supplement: Table S3 — Results of principal components analysis performed with six variables (TB antigen-induced cytokine/chemokine results selected to discriminate between latently infected adults and negative controls). Components with Eigenvalues >1 are shown. (0.04 MB DOC) [file pone.0008804.s003.doc]

**Supplemental Table S3**

**Table S3. *Results of principal components analysis* performed with six variables** (TB antigen-induced cytokine/chemokine results selected to discriminate between latently infected adults and negative controls). Components with Eigenvalues >1 are shown. The correlation of a variable with each component is derived as the product of the eigen coefficient and the square root of the eigenvalue).

| Parameters | 1^st^ Component  (“Th-1- like”) | 2^nd^ Component  (“Th-2-like”) |
| --- | --- | --- |
| Eigenvalue  % Variation explained | 2.638  44 | 1.51  25 |
| *Coefficients* |  |  |
| IFN-γ | 0.53 | -0.29 |
| IL-2 | 0.48 | -0.32 |
| CXCL-10 (IP10) | 0.43 | -0.08 |
| TNF-α | 0.36 | -0.08 |
| IL-13 | 0.33 | 0.60 |
| IL-5 | 0.26 | 0.66 |
