# Supplementary material for: Infection with Helicobacter pylori Is Associated with Protection against Tuberculosis
Source: PLoS One. 2010 Jan 20;5(1):e8804. doi: 10.1371/journal.pone.0008804 (PMC2808360; doi:10.1371/journal.pone.0008804)
Supplement: Table S4 — Baseline characteristics of human cohort samples. LTBI: latent tuberculosis infection determined by TST ≥10mm and/or ELISPOT ≥8 SFU (Gambia) or TST ≥10 mm (Pakistan). (0.04 MB DOC) [file pone.0008804.s004.doc]

**SupPLEMENTAL Table S4**

**Table S4**. Baseline characteristics of human cohort samples

| **Characteristic** | **Gambia** | **Pakistan** | *p-value* |
| --- | --- | --- | --- |
| Eligible samples | 2626 | 121 |  |
| Number sampled (sampling proportion) | 549 (22) | 121 (100) |  |
| Households, # | *271* | *20* |  |
| Age, mean (range) | 27 (3-100) | 26 (6-70) | *0.43* |
| Child, # <18y (%) | 142 (26) | 44 (36) | *0.02* |
| Sex, # male (% male) | 245 (45) | 52 (43) | *0.74* |
| LTBI, # (%) | 372 (68) | 94 (78) | *0.03* |
| *Reported cohort activation rate @ 24 mos* | 1.1% [1] | 6.4% [2] |  |

LTBI: latent tuberculosis infection determined by TST≥10mm and/or ELISPOT ≥8 SFU (Gambia) or TST ≥10 mm (Pakistan)
