# Supplementary material for: Infection with Helicobacter pylori Is Associated with Protection against Tuberculosis
Source: PLoS One. 2010 Jan 20;5(1):e8804. doi: 10.1371/journal.pone.0008804 (PMC2808360; doi:10.1371/journal.pone.0008804)
Supplement: Table S5 — Seroprevalence of H. pylori CagA infection in TB cases and household contacts: differences between The Gambia and Pakistan. (0.03 MB DOC) [file pone.0008804.s005.doc]

**SupPLEMENTAL tABLE s5**

**Table S5: Seroprevalence of *H. pylori* CagA infection in TB cases and household contacts: differences between The Gambia and Pakistan.**

| **Clinical Definition/Cohort** | **Gambia**  **(n=549)** | **Pakistan**  **(n=121)** | *p-value* | **Total**  **(n=670)** |
| --- | --- | --- | --- | --- |
| **TB case (prevalent)** | 51(51) | 8 (40) | *0.37* | 59 (49) |
| **TB case (incident)** | 24 (83) | 3 (38) | *0.02* | 27 (73) |
| **Nonprogressor** | 291 (69) | 48 (52) | *0.001* | 339 (66) |
| **Total** | 366 (67) | 59 (49) | *0.0002* | 425 (63) |
