# Supplementary material for: Infection with Helicobacter pylori Is Associated with Protection against Tuberculosis
Source: PLoS One. 2010 Jan 20;5(1):e8804. doi: 10.1371/journal.pone.0008804 (PMC2808360; doi:10.1371/journal.pone.0008804)
Supplement: Table S6 — Factors associated with H. pylori CagA infection in Gambia and Pakistan tuberculosis case-contact cohort samples (univariate analysis). (0.04 MB DOC) [file pone.0008804.s006.doc]

**SupPLEMENTAL tABLE S6**

**Table S6. Factors associated with *H. pylori* CagA infection in Gambia and Pakistan tuberculosis case-contact cohort samples (*univariate analysis*)**

| **Factor** | **Reference** | **Odds Ratio**  **(95% CI)** | ***p-value*** | **Odds Ratio**  **(95% CI)** | ***p-value*** |
| --- | --- | --- | --- | --- | --- |
|  | **Gambia** |  |  | **Pakistan** |  |
| Age | 10 y Δ | 0.83 (0.74-0.92) | *0.001* | 0.94 (0.74-1.2) | *0.68* |
| Sex | Female | 0.93 (0.65-1.33) | *0.69* | 1.1 (0.53-2.5) | *0.83* |
| LTBI | Uninfected | 0.77 (0.52-1.13) | *0.19* | 0.31 (0.12-0.78) | *0.01* |
| TB Outcome group | Nonprogressor |  |  |  |  |
|  | Index TB Case | 0.46 (0.30-0.72) | *0.001* | 0.63 (0.22-1.7) | *0.35* |
|  | New TB case | 2.0 (0.76-5.5) | *0.166* | 0.56 (0.11-2.4) | *0.45* |
