# Supplementary material for: Infection with Helicobacter pylori Is Associated with Protection against Tuberculosis
Source: PLoS One. 2010 Jan 20;5(1):e8804. doi: 10.1371/journal.pone.0008804 (PMC2808360; doi:10.1371/journal.pone.0008804)

**Supporting Information**

**Figure S1. Measles antibody responses in 100 Gambian TB cases and 100 latently infected household contacts**


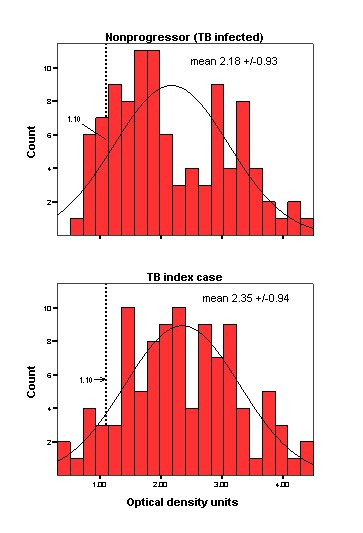

Supplement: Figure S1 — Measles antibody responses in 100 Gambian TB cases and 100 latently infected household contacts. Ref. line: positive cut-off (Bioquant IgG ELISA, San Diego, CA); Nonprogressor, household contact of TB index case remaining disease-free for at least 2 years from baseline; TB infected, positive TST ≥10mm or ELISPOT ≥8 SFU at baseline. (0.04 MB DOC) [file pone.0008804.s007.doc]
